# Removal of GABA_A_ Receptor γ2 Subunits from Parvalbumin Neurons Causes Wide-Ranging Behavioral Alterations

**DOI:** 10.1371/journal.pone.0024159

**Published:** 2011-09-02

**Authors:** Elli Leppä, Anni-Maija Linden, Olga Y. Vekovischeva, Jerome D. Swinny, Ville Rantanen, Esko Toppila, Harald Höger, Werner Sieghart, Peer Wulff, William Wisden, Esa R. Korpi

**Affiliations:** 1 Institute of Biomedicine, Pharmacology, University of Helsinki, Helsinki, Finland; 2 Medical Research Council, Anatomical Neuropharmacology Unit, Department of Pharmacology, Oxford University, Oxford, United Kingdom; 3 Institute for Biomedical and Biomolecular Sciences, School of Pharmacy and Biomedical Sciences, University of Portsmouth, Portsmouth, Hampshire, United Kingdom; 4 Research Programs Unit, Genome-Scale Biology, and Institute of Biomedicine, Biochemistry and Developmental Biology, University of Helsinki, Helsinki, Finland; 5 Finnish Institute of Occupational Health, Helsinki, Finland; 6 Core Unit of Biomedical Research, Division of Laboratory Animal Science and Genetics, Medical University Vienna, Himberg, Austria; 7 Department of Biochemistry and Molecular Biology, Center for Brain Research, Medical University Vienna, Vienna, Austria; 8 Department of Clinical Neurobiology, University of Heidelberg, Heidelberg, Germany; Tokyo Medical and Dental University, Japan

## Abstract

We investigated the behavioral significance of fast synaptic inhibition by αβγ2-type GABA_A_ receptors on parvalbumin (Pv) cells. The GABA_A_ receptor γ2 subunit gene was selectively inactivated in Pv-positive neurons by Cre/loxP recombination. The resulting Pv-Δγ2 mice were relatively healthy in the first postnatal weeks; but then as Cre started to be expressed, the mice progressively developed wide-ranging phenotypic alterations including low body weight, motor deficits and tremor, decreased anxiety levels, decreased pain sensitivity and deficient prepulse inhibition of the acoustic startle reflex and impaired spatial learning. Nevertheless, the deletion was not lethal, and mice did not show increased mortality even after one year. Autoradiography with *t*-butylbicyclophosphoro[^35^S]thionate suggested an increased amount of GABA_A_ receptors with only α and β subunits in central nervous system regions that contained high levels of parvalbumin neurons. Using BAC-transgenesis, we reduced some of the Pv-Δγ2 phenotype by selectively re-expressing the wild-type γ2 subunit back into some Pv cells (reticular thalamic neurons and cerebellar Pv-positive neurons). This produced less severe impairments of motor skills and spatial learning compared with Pv-Δγ2 mice, but all other deficits remained. Our results reveal the widespread significance of fast GABAergic inhibition onto Pv-positive neurons for diverse behavioral modalities, such as motor coordination, sensorimotor integration, emotional behavior and nociception.

## Introduction

Parvalbumin (Pv)-positive neurons, with their diverse morphologies and firing patterns, contribute to neuronal network function throughout the central nervous system [1], [2], [3], [4], [5]. Disturbances in Pv cell activity in particular brain areas, such as frontal cortex, hippocampus or cerebellum, can lead to dysfunctional information processing [6], [7], [8], [9], [10], [11], [12], [13]. Mostly, Pv-positive neurons release GABA, either as interneurons (e.g. cortical basket or axo-axonic cells) or projection neurons (e.g. cerebellar Purkinje cells or reticular thalamic neurons). In all cases GABAergic Pv cells, because they themselves receive inhibition from each other and other types of GABA neurons, participate in complex loops of feed-forward and feedback inhibition [6], [14]; a minority of Pv cells, however, are glutamatergic e.g. some cortico-striatal projection neurons [15], [16], [17]. Some brain regions, such as the cerebellar cortex and the reticular thalamic nucleus, contain mostly or only Pv-positive cells; in other brain areas, such as the caudate-putamen, colliculi, deep cerebellar nuclei, neocortex and hippocampus, Pv cell types form a minority, but they are important for organizing general network activity [14].

Given the ubiquity of both GABAergic transmission in the CNS and the expression of the GABA_A_ receptor γ2 subunit gene [18], [19], it seems likely that most, if not all, Pv cell types receive fast synaptic inhibition via αβγ2 type GABA_A_ receptors. Within such a typical GABA_A_ receptor complex, the γ2 subunit allows allosteric modulation of GABA_A_ receptor complexes by drugs such as benzodiazepines and β-carbolines [18]. More fundamentally, since the γ2 subunit targets the GABA_A_ receptor to the synapse and provides the full single channel conductance [20], [21], [22], knock-out of the γ2 subunit ablates fast synaptic inhibition [6], [11], [12], [23] with the remaining αβ GABA_A_ receptors providing receptors of low conductance and short open duration [21], [24]. Indeed, the functions of αβγ2 GABA_A_ receptors are so essential for CNS circuitry, that total knockout of the γ2 subunit is lethal in the early postnatal period [25]. Even without a total knockout, if the level of γ2 protein is reduced (heterozygote γ2 knockout or neo gene insertion into an intron) neurological and behavioral phenotypes appear [26], [27], [28]. The haplo-insufficiency implies that healthy neurons normally have sufficient, but not excess, γ2 protein levels.

In contrast to the γ2 gene total knockouts, cell-type-selective inactivations of the γ2 subunit gene give, as expected, a range of network and behavioral phenotypes, some major and lethal, some subtle, depending on cell type and age [6], [11], [12], [22], [29], [30]. Furthermore, removing γ2 from neurons is not necessarily excitatory in the sense that it makes the cells fire more action potentials; Purkinje neurons, for example, have an intrinsic pacemaker. Removing γ2 and fast synaptic inhibition from these Pv-positive cells makes them fire action potentials more regularly, but counter-intuitively, they do not fire more (see [12]). Finally, dominant negative mutations (R43Q) in the γ2 subunit gene can produce epilepsy in human neonates and adults [31], [32].

In Pv-Δγ2 mice, the γ2 subunit gene was inactivated via Cre/loxP recombination in Pv-neurons [6]. In electrophysiological recordings from Pv-Δγ2 mice fast IPSCs are missing from hippocampal Pv-interneurons. Similarly, in cerebellar Purkinje cell-Δγ2 mice (PC-Δγ2) fast IPSCs are missing from Purkinje cells after removal of the γ2 subunit; only small, slow inhibitory currents remain, which may be due to spillover of GABA from the synapse to low conductance extrasynaptic αβ subunit-containing receptors [6], [11], [12]. In Pv-Δγ2 mice, the lack of fast synaptic inhibition onto Pv-neurons disrupts hippocampal network oscillations: theta band oscillations are reduced several fold in power and have reduced frequency, and although gamma band oscillations are intact with respect to frequency and power, the coupling between theta and gamma band oscillations is disrupted [6]. In PC-Δγ2 mice, Purkinje cells show altered firing patterns and the mice cannot consolidate new motor memories [12].

In this article, we describe the phenotype of the Pv-Δγ2 mice at the behavioral level and correlate these changes with those found for GABA_A_ receptor binding sites in specific brain regions. We also restored the wild-type γ2 subunit to some Pv-cell types of Pv-Δγ2 mice such as the cerebellar molecular layer neurons and reticular thalamus (Pv-Δγ2-partial rescue mouse line). This enabled us to judge which parts of the behavioral phenotype of Pv-Δγ2 mice were likely due to disturbances in other brain regions beyond the cerebellum and reticular thalamus.

## Methods

### Animals

#### Ethics

All procedures for generation and maintenance of mouse lines were done in accordance with the United Kingdom Animals (Scientific Procedures) Act 1986 (Home Office Licence number **PPL 60/3562)**, and had ethical approval from the Tierschutz Commission of the Regierungspraesidium Karlsruhe, Germany (project title “veraenderte Ionenkanaele im Gehirn”, granted 30.09.2002). All behavioral animal experiments were carried out with the permissions (ESLH-2004-01605/Ym-23 and ESLH-2006-09005/Ym-23) of the State Provincial Government of Southern Finland. All efforts were made to minimize the number and suffering of animals.

#### γ2I77lox and Pv-Δγ2 mice

As a background line we used γ2I77lox mice [11], in which the GABA_A_ receptor γ2 subunit F77 residue is point-mutated to encode I77, causing an inability of the γ2 subunit-dependent benzodiazepine binding site to bind the sedative-hypnotic zolpidem and the β-carboline convulsant 3-carbomethoxy-4-ethyl-6,7-dimethoxy-β-carboline (DMCM) [33], [34]. In other aspects the γ2I77 subunit-containing GABA_A_ receptors function normally [34]. Pv-Δγ2 mice were generated by crossing γ2I77lox mice with PvCre mice as previously described [6].

#### Pv-Δγ2-partial rescue mice

**Figure 1:**
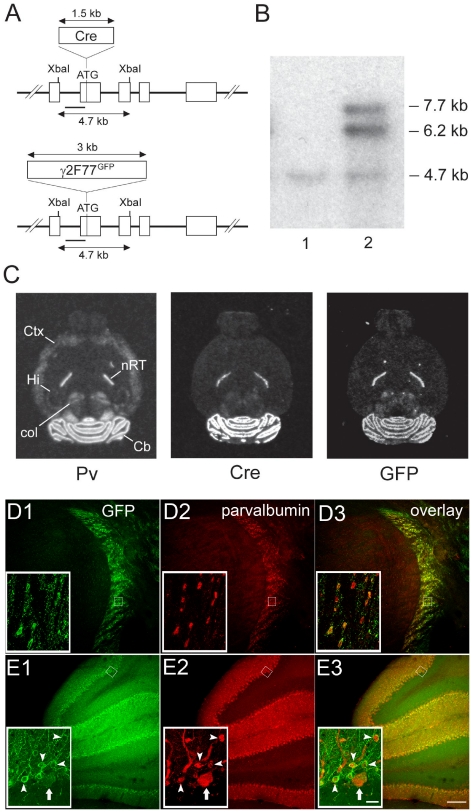
Construction of Pv-Δγ2-partial rescue mice. (**A**) Illustration of the PvBAC-Cre and PvBAC-γ2F77^GFP^ transgene constructs. Cre recombinase and γ2F77^GFP^ cDNAs respectively were inserted into the start codon located in exon 2 of the Pv gene in BAC 450D23 via bacterial homologous recombination. XbaI restriction sites and the probe (black bar) used for southern blot analysis of founder mice are indicated. (**B**) Southern blot analysis of XbaI-digested genomic DNA of a wild-type (lane 1) and a transgenic (lane 2) animal hybridized with the 5′ recombinogenic arm shows bands at about 4.7 kb (wild-type), 6.2 kb (Cre transgene) and 7.7 kb (γ2F77^GFP^ transgene). (**C**) *In situ* hybridization autoradiographs show the expression of Cre (middle panel) and γ2F77^GFP^ (right panel) mRNA in Pv-Δγ2-partial rescue mice, which is less widespread than native expression of Pv mRNA (left panel). *Cb*, cerebellum; *col*, superior and inferior colliculi; *Ctx*, cortex; *Hi*, hippocampus; *nRT*, reticular nucleus of thalamus. (**D**) Overview of GFP and parvalbumin immunoreactivity in the thalamus with magnified views provided by the inserts. D1 shows that GFP labelling is restricted to the reticular nucleus of the thalamus. D2 shows parvalbumin labelling only in neurons of the reticular nucleus. D3, an overlay of D1 & D2 shows the co-localisation of GFP with parvalbumin confirming the restricted expression of GFP only in parvalbumin-containing neurons in this brain region. (**E**) Overview of GFP and parvalbumin immunoreactivity in the cerebellum with magnified views provided by the inserts. **E1** shows that GFP labelling is concentrated in the Purkinje cell and molecular layers of the cerebellar cortex with the insert showing strong immunoreactivity on Purkinje cell dendrites and molecular layer interneurons (arrowheads) with weaker labeling evident in a Purkinje cell soma (arrow). **E2** shows characteristic parvalbumin labelling restricted to Purkinje cells and molecular layer interneurons (arrowheads). **E3**, an overlay of E1 & E2 shows the co-localisation of GFP with parvalbumin confirming the restricted expression of GFP only in parvalbumin-containing neurons in the cerebellum. Scale bar 200 µm, insert 20 µm.

If two different transgenes are mixed and co-injected into pro-nuclei, they often co-integrate in the same genomic position (e.g.[35], [36]). We exploited this to link Cre and γ2F77^GFP^ transgene expression in Pv cells in the same mouse line by co-injecting two Pv BAC transgenes: PvCre [9] and Pvγ2F77^GFP^. To make the Pvγ2F77^GFP^ transgene, we followed the strategy of [4] and [9], who placed eGFP and Cre respectively into the first coding exon of the Pv gene via bacterial homologous recombination (Figure 1A). We used BAC clone 450D23 from the mouse 129SV strain library (Research genetics, Inc., Huntsville, AL, USA) containing the Pv gene plus ≥50 kb upstream and 15 kb downstream genomic sequence [4], [9].The targeting cassette consisted of a reading frame of γ2L fused at its N-terminus with an eGFP/9E10 epitope tag (γ2F77^GFP^) [11], [37] flanked by stretches of homologous sequence upstream and downstream of the translational start of the Pv gene. The 3′ recombinogenic arm (612 bp) was generated previously by PCR with the following primers: Pv3′RA_s (5′-GCCTTTGCTGGTGAGCAATGCAC-3′) and Pv3′RA_as (5′-AAGAGATCACACAGCCGAGTGGGT-3′), and inserted into the EcoRV site of pBluescript II SK to generate pBS-3′RA [4]. The γ2F77^GFP^ reading frame was inserted into an EcoRI site immediately 5′ of the 3′ recombinogenic arm in pBS-3′RA to generate pBS-γ2F77^GFP^-3′RA. The 5′ recombinogenic arm (1.2 kb) containing a SalI site at the 5′ end was generated by PCR with the following primers: Pv5′RA_s (5′-CCCCGTCGACCAGGGCTCAGCTAAGGAA-3′), Pv5′RA_as (5′-CTGCAACTGTTTGAGCGGGCAGAG-3′). To fuse the 5′ untranslated region of the Pv gene directly to the translational start of γ2F77^GFP^, a 280 bp fragment of the 5′ coding sequence of γ2F77^GFP^ was amplified by PCR using the following primers: γ2^GFP^s (5′-G**ATG**AGTTCGCCAAATACATGGA-3′) and γ2^GFP^as (5′-GGTGGTACCGATGAACTTCAGG-3′). A KpnI site was introduced into the 3′ end of the PCR product during amplification. The 5′ recombinogenic arm was cut with SalI, the 280 bp PCR product of γ2F77^GFP^ was cut with KpnI. The blunt ends of these fragments were joined in a triple ligation into pBluescript II SK digested with SalI and KpnI to generate pBS-5′RA-5′γ2F77^GFP^. The 5′RA-5′γ2F77^GFP^ insert was released from pBS-5′RA-5′γ2F77^GFP^ by BglII/EcoRI digest and inserted into pBS-γ2F77^GFP^-3′RA digested with BglII and partially digested with EcoRI to result in pBS-5′RA-γ2F77^GFP^-3′RA. The final recombination cassette containing the 5′ and 3′ recombinogenic arms flanking the γ2F77^GFP^ reading frame was released from pBS-5′RA-γ2F77^GFP^-3′RA by SalI digest and inserted into the SalI-digested shuttle vector pSVrecA. The recombination cassette was integrated into the translational start of the Pv gene in BAC 450D23 via bacterial homologous recombination [38]. For pronucleus injection, BAC DNA was prepared by cesium chloride gradient centrifugation and digested with NotI to release the BAC insert [4]. The insert was separated from the vector on a CL4B-Sepharose (GE Healthcare Life Sciences Ltd, UK, Amersham Place, Buckingshamshire, UK) column.

For the co-injection of the PvBAC-γ2F77^GFP^ with the PvBAC-Cre transgenes [9], the two isolated BAC inserts were mixed 1:1 prior to injection. The mixture was injected into the pronuclei of B6D2F2 mouse zygotes at a concentration of 0.7 µg/ml (done by Dr. F. Zimmermann, University of Heidelberg). Founder mice were identified by Southern blot analysis of XbaI digested genomic DNA hybridized with the 5′ recombinogenic arm. This probe recognizes a ∼4.7 kb wild-type band, a ∼6.2 kb band in mice transgenic for Cre recombinase and a ∼7.7 kb band in mice transgenic for γ2F77^GFP^ (see Figure 1B). The same method was used for genotyping the offspring. Brains of offspring were analysed by *in situ* hybridization (see below) with eGFP- and Cre-specific probes, which pick out the γ2F77^GFP^ and Cre transgenes, respectively (Figure 1). Three lines showed transgene expression. One line (PC7) was then crossed with γ2I77lox mice [6], [11] to generate Pv-Δγ2-partial rescue mice.

### Neurochemical assays

#### *In situ* hybridization

*In situ* hybridization to adult mouse brain sections with [^35^S]-labeled oligonucleotide probes was performed as described [39]. Images were generated from 10-week exposures to Kodak Biomax MR X-ray film (Eastman Kodak, Rochester, NY). To assess non-specific labeling of the sections, each labeled oligonucleotide was hybridized to brain sections with a 100-fold excess of unlabeled oligonucleotide. Oligonucleotide sequences were:

eGFP:

5′-ATGCGGTTCACCAGGGTGTCGCCCTCGAACTTCACCTCGGCGCGGGT-3′,

Cre recombinase:

5′-CTGAACATGTCCATCAGGTTCTTGCGAACCTCATCACTCGTTGCA-3′,

Pv:

5′-TCTTCAGGCCCACCATCTGGAAGAACTTTTTGTGGTCGAAGGAGT-3′.

#### Immunohistochemistry

To confirm the selective expression of GFP in a subset of PV-containing neurons, *Pv-Δγ2-partial rescue* mice were deeply anaesthetized with sodium pentobarbital (100 mg/kg, i.p.) and transcardially perfused in accordance with the UK Animals (Scientific Procedure) Act 1986 and associated procedures. The initial solution was 0.1 M phosphate-buffered saline (PBS), followed for 12 min by a fixative composed of 4% paraformaldehyde and 0.2% picric acid made up in 0.1 M phosphate buffer (PB, pH 7.2). Brains were removed, and sectioned in the sagittal plane on a Vibratome. Double immunofluorescence reactions were performed on free-floating sections. These were incubated in a blocking solution of 20% normal donkey serum (NDS) diluted in Tris-buffered saline (TBS, pH 7.4, 0.3% Triton) for 2 h. The sections were then incubated in the following cocktail of primary antibodies overnight at 4°C: affinity purified rabbit anti-GFP (1∶1000) (Invitrogen; Catalog Number A-11122), and mouse anti-parvalbumin (1∶5000) (Swant; Catalog Number 235). The following day, the sections were rinsed thoroughly in TBS and then incubated in a cocktail containing donkey anti-rabbit Alexa 488 (1∶1000) (Invitrogen) and donkey anti-mouse Cy3 (1∶1000) (Jackson ImmunoResearch) for 2 h at room temperature. The sections were rinsed in PB and mounted in Vectashield (Vector Laboratories, Burlingame, CA).

Sections were examined with a confocal laser-scanning microscope (LSM710; Zeiss, Oberkochen, Germany) using either a Plan Apochromatic 63× DIC oil objective (NA1.4). Z-stacks were used for routine evaluation of the labelling. The images presented represent a single optical section. These images were acquired using sequential acquisition of the different channels to avoid cross-talk between fluorophores, with the pinholes adjusted to one airy unit for all channels. Images were processed with the software Zen2008 Light Edition (Zeiss) and exported into Adobe Photoshop. Only brightness and contrast were adjusted for the whole frame, and no part of a frame was enhanced or modified in any way.

#### Ligand autoradiography

To assess the expression of wild-type γ2 subunits forming high-affinity GABA_A_ receptor benzodiazepine binding sites from the Pvγ2F77^GFP^ transgene in Pv-Δγ2-partial rescue mice, [^3^H]Ro 15-4513 autoradiography was performed as described [40]. Fourteen-µm cryostat sections were obtained from wild-type γ2F77 (n = 2), γ2I77 (n = 7), Pv-Δγ2 (n = 5) and Pv-Δγ2-partial rescue (n = 7) mice. Brain sections were preincubated for 15 min in an ice-water bath in incubation buffer [50 mM Tris-HCl, 120 mM NaCl (pH 7.4)]. The final incubation took place with 15 nM [^3^H]Ro 15-4513 (Perkin Elmer, Boston, MA, USA) in the incubation buffer in the dark at 4°C for 60 min in plastic slide mailers. Nonspecific binding was determined with 10 µM Ro 15-1788 (flumazenil; Tocris Biosciences, Ellisville, MO, USA). After the incubation the sections were washed with ice-cold incubation buffer for 2×60 s, dipped in distilled water and dried in air flow at room temperature.

*t*-Butylbicyclophosphoro[^35^S]thionate ([^35^S]TBPS) binding to mouse brain sections was performed as described [41]. Sections from γ2I77 (n = 6), Pv-Δγ2 (n = 5) and Pv-Δγ2-partial rescue (n = 6) mice were preincubated 3×10 min in an ice-water bath in 50 mM Tris-HCl (pH 7.4) in the presence of 1 mM EDTA (Sigma Aldrich Chemical Company, St. Louis, MO, USA), which was then washed out. The final incubation took place with 6 nM [^35^S]TBPS (GE Healthcare corp., Piscataway, NJ, USA) in the incubation buffer (50 mM Tris-HCl, 120 mM NaCl, pH 7.4) at room temperature for 90 min. Nonspecific binding was determined with 100 µM picrotoxinin (Sigma). The effects of 2 µM and 1 mM GABA on radioligand binding were investigated. After the incubation the sections were washed with buffer (10 mM Tris-HCl, 120 mM NaCl, pH 7.4) for 3×2 min (for 2 µM GABA) or 3×30 min (for 1 mM GABA), dipped in distilled water and dried in air flow at room temperature.

For [^3^H]Ro 15-4513 autoradiography, the sections were exposed to Kodak Biomax MR film for 24 weeks with ^3^H-radioactivity standards (GE Healthcare); for [^35^S]TBPS autoradiography, sections were exposed to the same film type for 6 weeks with ^14^C-radioactivity standards (GE Healthcare). Binding densities in the relevant brain areas were quantified with MCID M5-imaging software (GE Healthcare) and converted to radioactivity values (nCi/mg for ^3^H and nCi/g for ^14^C) on the basis of the simultaneously exposed standards. Nonspecific binding was subtracted from all values.

### Behavioral experiments

Animals were bred either in the University of Heidelberg, Germany, in the University of Aberdeen, UK, or in the Medical University Vienna, Austria and sent to Helsinki at the age of 4–5 months, after which they were acclimatized to the animal facility conditions for 7 days before commencing behavioral testing. The animals were housed (1–5 per cage) in transparent polypropylene cages (37×21×15 cm, Tecniplast, Buguggiate, Italy) with standard rodent pellets (Harlan BV., Horst, Netherlands) and tap water *ad lib*. Lights were on from 7 a.m. to 7 p.m., under 12∶12 h light∶dark cycles, at 21–23°C and a humidity of 50–60%.

Pv-Δγ2 male (n = 16) mice, Pv-Δγ2-partial rescue male (n = 16) and female (n = 20) mice, littermate wild-type γ2F77 male (n = 3) and female (n = 2) and control littermate γ2I77 male (n = 31) and female (n = 16) mice aged 4–10 months (15–45 g) were used.

The SHIRPA observational screen was performed with naïve mice, after which elevated plus maze, acoustic startle and prepulse inhibition, Morris water maze, motor coordination, tremor and nociception testing were carried out. About one week after the last experiments the animals were strongly sedated with CO_2_, decapitated, and brains were collected for further analysis.

#### SHIRPA test

Basic behavioral and physiological characterization of phenotype was performed using a modified version [42] of the primary observational screen described in the SHIRPA protocol (http://empress.har.mrc.ac.uk/browser/?sop_id=10_002_0). The person who observed and recorded the behavior was blind to the genotype of the animals. The animals were naïve to handling at the time of SHIRPA testing.

#### Elevated plus-maze

To investigate the basal anxiety level of the mice, an elevated plus-maze test was performed. The test was performed after the primary SHIRPA screen, before any other tests. The apparatus was made of grey plastic and elevated to 50 cm from the floor level. It consisted of a central platform (5×5 cm), from which two open arms (5×40 cm with a 0.7 cm ledge) and two enclosed arms (5×40×20 cm) extended [43]. The light intensity was set at 50 lux on the open arms and 20 lux in the closed arms. The mice were placed individually on the central platform facing an open arm and allowed free exploration of the maze for 5 min with their behavior being recorded using a video tracking system with a CCD video camera above the plus maze [44]. The position and movements of the center of the animal's surface area were analyzed automatically using EthoVision software (Noldus Information Technology, Wageningen, Netherlands). The central area was extended to include the first 2 cm of each arm. An arm entry was recorded when the center of the mouse entered the distal part of the arm. This corresponds to the definition of an arm entry with all four legs on the arm [43]. The plus-maze was cleaned with a water-moistened paper towel and dried after each mouse. The mice were returned to their home cage when all mice from the same cage were tested.

#### Acoustic startle response and prepulse inhibition

The acoustic startle response and prepulse inhibition (PPI) were measured using a two-unit automated startle system (Startle Reflex System, Med Associates Inc., St. Albans, VT, USA) based on previously described procedures for mice (modified from [45], [46]). In an illuminated and sound-attenuated chamber, a small cage (7.5 cm long, 3.5 cm wide and 4.0 cm high) with metal bars was mounted above a piezoelectric sensor. Movements of the animal in the cylinder were detected by a piezoelectric sensor, digitized and analyzed by Startle Reflex System software (version 4.01, Med Associates Inc.). The sensitivities of the two chambers were calibrated and adjusted to be identical (Platform calibrator, Med Associates Inc.). Background noise of 65 dB and acoustic stimuli were delivered through speakers in the ceiling of the chambers. Mice were acclimated to the cylinders and chambers for 5 min daily for five days before the experiments. First, acoustic startle responses to stimuli of different intensities were determined. The test session began with a 5-min acclimation. Background noise was on during the acclimation period and throughout the session. The acclimation period was followed by seven blocks of trials containing seven stimuli (40 ms) of different intensities (65, 71, 75, 85, 95, 110 and 120 dB) in a pseudorandom order. The intertrial interval varied between 9–21 s. The test session contained 49 trials and lasted approximately 20 min.

Following at least a 3-day wash-out after startle testing, PPI was analyzed. To habituate the mice to the test stimuli, the test session began with six trials of 110 dB stimuli (40 ms) which were not included in the analysis. This was followed by 10 blocks of four trials containing 110 dB startle stimuli alone and combined to a 71 dB prepulse stimulus (20 ms). The prepulse stimulus was also delivered alone five times during the session. The order of different type of stimuli was pseudorandom. The interval between prepulse and startle stimuli was 100 ms and intertrial interval varied between 9–21 s. The test session contained 51 trials and lasted approximately 20 min. To investigate whether antipsychotics could reverse the observed deficits in PPI, the effects of 0.5 mg/kg haloperidol (20 min before testing) [47] and 4 mg/kg clozapine (30 min before testing) [46] were tested in Pv-Δγ2-partial rescue mice. To investigate whether psychotomimetics would deteriorate the deficits further, Pv-Δγ2-partial rescue mice were administered 0.15 mg/kg MK-801 (20 min before testing) [48].

The movements of the mouse were sampled for 50 ms before the first stimulus (null period) and for 200 ms after the first stimulus. The startle amplitude was defined as the peak amplitude that occurred during the first 100 ms after the onset of the startle stimulus. If the movements of the animal produced a clear signal (differed more than±150 units from zero) during the null period, the trial was excluded from the results. The exclusion was done by an experimenter viewing graphs from each trial without knowing the type of the stimulus and blind to animal genotype. The percent prepulse inhibition (PPI) was calculated from the formula: %PPI = [(amplitude of startle pulse alone – amplitude of startle pulse when preceded by a prepulse)/amplitude of startle pulse alone]×100.

As an enhanced sensitivity to 0.15 mg/kg MK-801 was observed in the PPI experiments, behavioral scoring of Pv-Δγ2-partial rescue mice was conducted in a separate experiment. MK-801 (0.15 mg/kg) or vehicle was administered to Pv-Δγ2-partial rescue and control γ2I77 mice and their behavior was observed in the home cage at 5 min intervals for 20 min and following that at 60, 90, 120 and 180 min after injection. The mice were visually scored (±) for the presence of any abnormal symptoms (agitation, jumping, hyperlocomotion, stereotypic behavior, seizures).

#### Morris water maze

Mice were trained during 4 days (6 trials/day, with a 10-min inter-trial interval) to find a platform submerged circa 1 cm below the surface of a 120-cm diameter pool of water. Visual markers were placed on the walls of the experiment room to facilitate spatial learning. The pool was divided into 4 equal quadrants. During training the platform was constantly in the same quadrant of the maze, and the quadrant in which the mice were placed varied from trial to trial in a pseudorandom manner. The water temperature was 19–20°C and the water was opacified with milk powder to prevent the mice from seeing the platform. Inter-trial interval was circa 10 min and trial cut-off time 3 min. If the mouse did not find the platform during a trial, it was gently guided onto it. The mice were towel-dried after each trial and placed on a heater pad (38°C) for 1 min before returning them to home cage. The escape latency, swimming velocity and total distance were recorded via Ethovision. The mice were also monitored from a TV-screen in a separate room to prevent drowning. One day after training, on day 5, a 1-min probe trial was performed in which the platform was removed and the time the mice spent in the platform quadrant as well as the number of visits to the former platform position and the swimming velocity were recorded (modified from [49]).

#### Motor tests

To investigate motor coordination of Pv-Δγ2 and Pv-Δγ2-partial rescue mice, rotarod tests were performed [50]. The mice were trained during 6 days (4–6 trials per day) to stay on a rotating rod (diameter 4 cm, Rotamex 4/8, Columbus Instruments, Ohio, USA) for 180 s, with the rotation speed being accelerated from 5 to 20 rpm (Pv-Δγ2 mice) or from 5 to 30 rpm (Pv-Δγ2-partial rescue mice). The latency to fall from the rod in each trial was recorded and a daily average of 4–6 trials was calculated for each animal.

The mice were also trained to walk along a 100-cm-long wooden beam (0.8 cm in diameter) back to their home cage [51]. One end of the beam was mounted on a supporter and the other on the edge of the home cage so that the beam was 84 cm above the floor. If the mouse did not move within 10 s it was gently pushed to induce movement. If the mouse slipped or fell it was helped back onto the beam. The latency to reach the other end of the beam was recorded. Training for the beam test was conducted twice a day for 6 days.

To assess pharmacological sensitivity, mice were injected with zolpidem (1–3 min prior to rotarod testing) or DMCM (both 15 min prior to rotarod testing). In some zolpidem and DMCM tests a pretreatment of 15 mg/kg flumazenil was used.

#### Tremor

Mice were habituated to glass holding jars (diameter 10 cm, height 14 cm) for 5 days, 5 min at a time. The tremor measurement apparatus was developed in-house and consisted of a piezoelectric weight sensor with three independent measuring stations. The holding jars were placed on fast scaling cells that measured weight up to 200 g. The measurement software was written using VEE Pro 6.0 (Agilent Technologies Inc., Espoo, Finland). The weight signal was sampled with an AD/convert (DT9804, Data Translation Inc., Marlboro, MA, USA) with a sample rate of 200 Hz and stored for further analysis. The tremor could be seen as fast variation in the weight signal. On the test day, baseline measurements were performed for 5 min, after which mice were injected with 15 mg/kg harmaline (Sigma) and tremor was measured 15 min later for 5 min. The data was analyzed with a software tool developed in-house, created with MathWorks Matlab® (MathWorks, Natick, MA, USA). From the total duration of all recorded tremor amplitude and frequency data, manually selected time regions were analyzed with a spectrogram function, to attain 1) the power of all possible frequencies found in the data at each time point, 2) the dominant frequency at each time point, which was calculated by finding which frequency had the maximum power at a given time and 3) dominant frequency distribution, which was calculated from the mean power of each frequency over the selected time period.

#### Pain sensitivity

Mice were acclimatized to the hot plate test apparatus (Hot Plate Analgesic Meter, Harvard Apparatus, Edenbridge, UK) for 5 days by placing them on an unheated hot plate inside a plastic cylinder (20 cm in diameter) daily for 3 min. During the test, the hot plate surface was maintained at 52±0.2°C. The latency to react was scored visually when the mouse rapidly moved or licked its hindpaw or jumped. The cutoff time was 40 s.

The mice were acclimatized to the tail-flick test procedure and apparatus (Model-DS20, Ugo Basile, Comerio, Italy) for 7 days by gently holding them immobile inside a cloth for 3 min. In testing basal pain sensitivity, the intensity of light was 15 V and the cutoff time 12 s. On the distal part of the tail, 1 cm area was marked with a black felt pen and heat was directed to this area. In testing for DMCM sensitivity, a shorter cut-off time of 8 s was used with slightly higher light intensity (24 V). Before drug or vehicle administration, two basal reaction times, 15 min apart, were determined. The latencies to the tail withdrawal reaction were analyzed after saline injection and 20 min after DMCM (3 mg/kg) injection.

#### Drugs for behavioral experiments

Zolpidem tartrate was crushed from tablets (Stilnoct®, Sanofi-Synthelabo AB, Bromma, Sweden) and suspended in physiological saline. DMCM (Sigma) was dissolved in physiological saline acidified with a few drops of 0.1 N HCl (pH>4). Flumazenil (Tocris Biosciences, Ellisville, MO, USA) was dissolved in Tween 80, which was diluted to 3% with physiological saline. Haloperidol was diluted from concentrate (Serenase®, Orion Pharma, Espoo, Finland) and brought to concentration with physiological saline. Clozapine was crushed from tablets (Leponex®, Novartis, Basel, Switzerland) and suspended in physiological saline. MK-801 (Sigma) was suspended in Tween 80 and brought to concentration with physiological saline (final Tween 80 concentration 3%). All substances were injected i.p. in a volume of 10 ml/kg.

### Statistical analyses

The results are given as means ± SEM, unless otherwise stated. Statistical tests were performed with SPSS Software (SPSS 12.0.1, SPSS Inc., Chicago, Illinois, USA) or GraphPad Prism software (Prism 5.0, GraphPad Software Inc., California, USA). Treatment groups and mouse lines were compared with either Student's *t*-test, Mann-Whitney U-test, Wilcoxon signed rank test, repeated measures ANOVA or one-way ANOVA followed by Newman-Keuls *post hoc*-test or Dunnett's test. In all statistical tests the level of significance was set at p<0.05.

## Results

### Mouse line description and general characterization

We used three strains of mice: the γ2I77lox line [11], [34], mice with selected disruption of the γ2 gene in Pv cells (Pv-Δγ2; [6]), and a novel strain (Pv-Δγ2-partial rescue), where the γ2 subunit is expressed in some Pv cell types but not others (Figure 1).

The origin of the Pv-Δγ2-partial rescue strain was accidental. In a similar way to how we made Purkinje cells selectively sensitive to zolpidem [11], [52], we had planned to make a complete swap of γ2 subunits in Pv cells, such that Pv cells would selectively express zolpidem-sensitive αβγ2F77^GFP^ GABA_A_ receptors and all other neurons in the brain would express the zolpidem-insensitive αβγ2I77 version. This swap would require deletion of the γ2I77 gene in Pv cells with Cre and simultaneous expression of γ2F77^GFP^ in the same Pv cells. To streamline the mouse breeding, we chose to co-integrate the PvCre and Pvγ2F77^GFP^ BAC transgenes. In these lines, we examined by *in situ* hybridization the neuronal expression of the BAC transgenes to see if they followed the expression of the endogenous Pv gene (Figure 1). Three founders (PC2, PC7, PC10) generated offspring with transgene expression, at the level of X-ray film analysis, that resembled the Pv gene expression “signature” of reticular thalamic and cerebellar molecular layer/Purkinje cell layer expression (compare the Cre, GFP and native Pv expression patterns seen by *in situ* hybridization; Figure 1C). The expression of Cre/γ2F77^GFP^ transgenes in other brain areas of Pv-Δγ2-partial rescue mice was negligible, except for a small amount in the superior and inferior colliculi (Figure 1C). Thus, the Cre/γ2F77^GFP^ transgene pattern did not fully resemble the native Pv gene expression pattern. Nevertheless, we took the line (PC7) with the strongest Cre and γ2F77^GFP^ transgene expression and crossed this line into the γ2I77lox line to generate, ultimately, the Pv-Δγ2-partial rescue mice: in the Pv-Δγ2-partial rescue mice, the γ2 gene is disrupted in most Pv-cell types, but γ2F77^GFP^ expression is present for example in the molecular layer cells of cerebellar cortex (Purkinje, stellate and basket cells) and the reticular thalamic neurons (Figure 1C-E).

**Figure 2:**
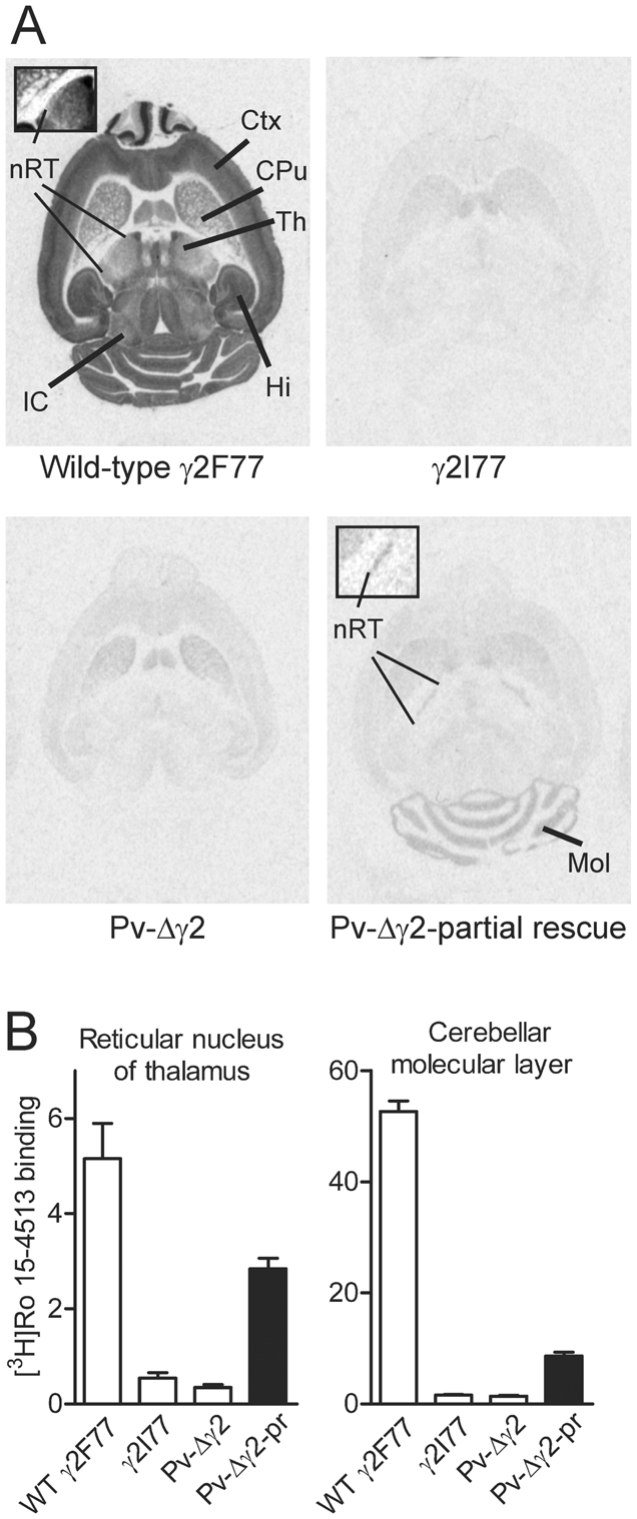
Autoradiographic distribution of brain GABA_A_ receptor benzodiazepine-site labeling in Pv-Δγ2 mouse models. (**A**) Representative autoradiographic images of high-affinity [^3^H]Ro 15-4513 binding to brain sections from wild-type γ2F77, control γ2I77, Pv-Δγ2 and Pv-Δγ2-partial rescue mice. *Ctx*, cortex; *CPu*, caudate-putamen; *Th*, thalamus; *nRT*, reticular nucleus of thalamus; *Hi*, hippocampus; *IC*, inferior colliculus; *Mol*, cerebellar molecular layer. Inserts depict the nRT in more detail. (**B**) Quantitative results of [^3^H]Ro 15-4513 binding to the reticular nucleus of thalamus and cerebellar molecular layer of wild-type γ2F77 (n = 2), control γ2I77 (n = 7), Pv-Δγ2 (n = 5) and Pv-Δγ2-partial rescue (n = 7) mice, indicating partial rescue of the binding sites in Pv-Δγ2-partial rescue mice. Data are presented as means ± SEM.

We used autoradiography of [^3^H]Ro 15-4513 binding to γ2 subunit-containing GABA_A_ receptor benzodiazepine sites [40] to confirm the pattern and amount of γ2F77^GFP^ transgene expression in Pv-Δγ2-partial rescue mice. Ro 15-4513 binds with high affinity to γ2F77 GABA_A_ receptors, but not to those with a γ2I77 mutation [34], [53], [54]. Binding in the cortex, thalamus, hippocampus, inferior colliculus and cerebellar granule cell layer of Pv-Δγ2-partial rescue mice was minimal compared with wild-type γ2F77 mice (Figure 2A for images), the small amount of residual binding being most likely due to GABA_A_ receptors with γ1 or γ3 subunits [55]. However, the [^3^H]Ro 15-4513 binding levels in Pv-Δγ2-partial rescue mice were approximately 55% of wild-type binding in the thalamic reticular nucleus and 16% in the molecular layer of cerebellum (Figure 2A–B). This matches the qualitative *in situ* hybridization data obtained with GFP probes (Figure 1C) and indicates rescue of pharmacologically active wild-type receptors in those brain regions.

### Altered GABA sensitivity and appearance of αβ-type GABA_A_ receptors in many brain areas of Pv-Δγ2 mice

**Figure 3:**
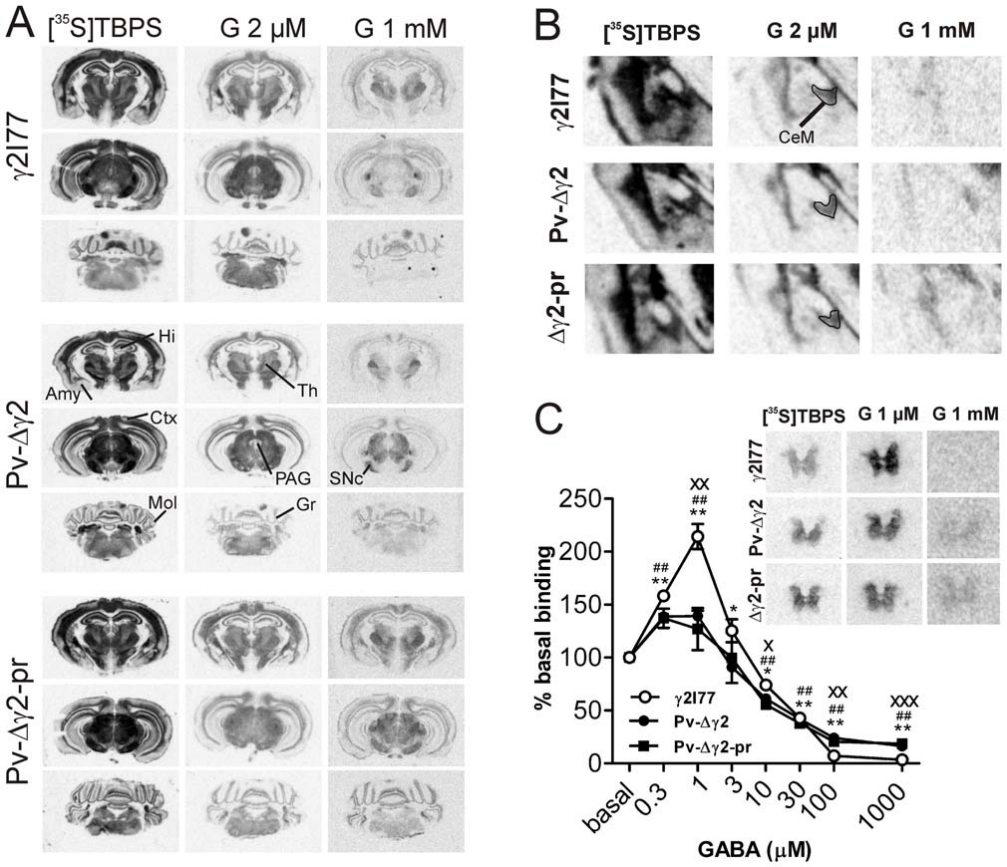
Characterization of brain regional GABA_A_ receptor ion channel binding sites and their GABA sensitivities in Pv-Δγ2 and control γ2I77 mouse lines. (**A**) Coronal autoradiographic images of [^35^S]TBPS binding to control γ2I77, Pv-Δγ2 and Pv-Δγ2-partial rescue mouse brain sections in the presence and absence of 2 µM and 1 mM GABA. The images for basal binding and the effects of 2 µM GABA were scanned with optimal scanning settings (similar for both conditions and for all mouse lines), whereas the images for the effects of 1 mM GABA were contrast enhanced for maximum clarity. *Ctx*, cortex; *Amy*, amygdala; *Hi*, hippocampus; *Th*, thalamus; *SNc*, substantia nigra pars compacta, *PAG*, periaqueductal gray area; *Gr*, cerebellar granule cell layer; *Mol*, cerebellar molecular layer. Section distances from bregma: forebrain −1.7, midbrain −3.16, cerebellum −5.68. (**B**) Detailed coronal images of [^35^S]TBPS binding to the amygdala regions from control γ2I77, Pv-Δγ2 and Pv-Δγ2-partial rescue mice (bregma −1.46), showing larger proportional reduction of binding by 2 µM GABA in the control and Pv-Δγ2-partial rescue mice than in Pv-Δγ2 mice. See, for quantitative results in Table S1). *CeM*, medial division of central amygdaloid nucleus. (**C**) Images of [^35^S]TBPS binding to control γ2I77, Pv-Δγ2 and Pv-Δγ2-partial rescue mouse spinal cord, demonstrating the loss of increase in binding by 1 µM GABA in the mutant as compared with the control γ2I77 mice. Also graphs presenting quantitative results of [^35^S]TBPS binding in spinal cord sections from control γ2I77 (n = 6), Pv-Δγ2 (n = 5) and Pv-Δγ2-partial rescue (n = 6) mice in the presence and absence of 0.3 µM−1 mM GABA are shown. The data are means ± SEM. * p<0.05, ** p<0.01 for the difference of the pharmacological effect in γ2I77 mice compared with baseline (repeated measures ANOVA and Newman-Keuls *post hoc* test). ^##^ p<0.01 for the difference of the pharmacological effect compared with baseline in Pv-Δγ2 and Pv-Δγ2-partial rescue mice. ^x^ p<0.05, ^xx^ p<0.01, ^xxx^ p<0.001 for the difference between γ2I77 and Pv-mouse lines.

Sections of Pv-Δγ2 (n = 5), Pv-Δγ2-partial rescue (n = 5) and control γ2I77 (n = 5) mouse brain and spinal cord were co-incubated with the GABA_A_ receptor ion channel ligand [^35^S]TBPS to visualize the overall density and GABA sensitivity of GABA_A_ receptors. The basal binding all over the brain and spinal cord was similar in all mouse lines, except for the midbrain and pons, in which several regions of the transgenic mice showed increased basal [^35^S]TBPS binding (Figure 3 and Table S1). However, the basal binding to cerebral cortex, striatum, hippocampus and cerebellum was not altered in the transgenic mice. The effect of 2 µM GABA, which is close to the GABA EC_50_ value, on [^35^S]TBPS binding was studied to evaluate the sensitivity of receptors, with the aim to localize αβ GABA_A_ receptors by reduced binding (more inhibition of binding by GABA) as γ2 subunit-deficient αβ receptors are known to be more sensitive to micromolar concentrations of GABA than αβγ2 receptors [24]. Changes in the effects to 2 µM GABA were detected widely in the midbrain and pontine structures, such as the substantia nigra pars reticulata, periaquaductal gray area, raphe nuclei and vestibular and olivary nuclei, in which both Pv-Δγ2 and Pv-Δγ2-partial rescue mice had larger percent inhibition of the binding than the control mice (Figure 3A and Table S1). Peculiarly, in some regions, such as the medial division of central amygdala of the Pv-Δγ2 mice, but not Pv-Δγ2-partial rescue mice, the opposite change was observed, i.e. 2 µM GABA affected the [^35^S]TBPS binding less in these mice than controls (Figure 3B, Table S1), the reason for which remains to be studied.

The effect of 1 mM GABA on [^35^S]TBPS binding was studied to further investigate the composition of the GABA_A_ receptors of Pv-Δγ2 and Pv-Δγ2-partial rescue mice. Changes in the amount of “GABA-insensitive binding”, i.e. [^35^S]TBPS binding which cannot be displaced by even high saturating concentrations of GABA, may imply changes in the ratio of αβγ2 receptors to αβ receptors [44], [56], [57]. This binding component was increased several fold in midbrain and pons of Pv-Δγ2 and Pv-Δγ2-partial rescue mice, and also widely in the forebrain areas of Pv-Δγ2-partial rescue mice (Figure 3A, Table S1). In many, but not all, of these areas, there were opposite changes from the control brains in the effects of 2 µM GABA and 1 mM GABA, which indicates that there was an increased population of GABA_A_ receptors without the γ2 subunit.

A more thorough concentration-response curve for GABA was established for the spinal cord sections from γ2I77, Pv-Δγ2 and Pv-Δγ2-partial rescue mice (Figure 3C). In the spinal cord of γ2I77 mice so-called “low-dose-hook effect” [58] was observed with GABA concentrations ranging from 0.3 to 3 µM, consistent with the selective non-equilibrium binding properties of α3 subunit-containing GABA_A_ receptors [59]. In the spinal cords of Pv-Δγ2 and Pv-Δγ2-partial rescue mice this effect of low GABA concentrations was attenuated. However, like in many midbrain and forebrain regions (Figure 3), the GABA-insensitive binding, determined in the presence of 1 mM GABA, remained higher in the Pv-Δγ2 and Pv-Δγ2-partial rescue mice than in the control mice (Figure 3C, Table S1).

### Characterization of the mice at the behavioral level

An observational screen was performed on Pv-Δγ2 (n = 15), Pv-Δγ2-partial rescue (n = 22) and γ2I77 control mice (n = 21) (Table 1). γ2I77 mice were previously found to be phenotypically similar to C57BL/6J and littermate control γ2F77 mice [34]. The Pv-Δγ2 and Pv-Δγ2-partial rescue mice showed no obvious behavioral alterations during the first postnatal weeks when compared with control γ2I77 mice (data not shown). However, as Cre started to be expressed from the Pv gene promoter during the second to third postnatal week [9] and no new γ2 protein was synthesized, Pv-Δγ2 and Pv-Δγ2-partial rescue mice began to show various phenotypical changes (see below), such that they were immediately recognizable from control mice. However, neither the Pv-Δγ2 nor Pv-Δγ2-partial rescue mice had an increased mortality relative to control mice by at least ca. one year of age (data not shown).

**Table 1:** Results of the SHIRPA screen on Pv-Δγ2 and Pv-Δγ2-partial rescue mice compared with control γ2I77 mice.

	Changes compared with control γ2I77 mice	
Parameter measured	Pv-Δγ2 mice	Pv-Δγ2-partial rescue mice
**body position**	0	0
**spontaneous activity**	−**	+***
**respiratory rate**	0	0
**tremor**	+***	+***
**urination**	0	0
**defecation**	0	−*
**locomotor activity**	0	+***
**startle**	+**	+***
**gait**	−*	0
**pelvic elevation**	−**	0
**tail elevation**	−***	0
**touch escape**	0	0
**trunk curl**	+***	+*
**limb grasping**	nm	+*
**visual placing**	0	0
**grip strength**	0	0
**pinna reflex, cornea reflex**	0	0
**toe pinch**	−*	0
**wire manoeuver**	−*	−***
**skin color**	0	0
**provoked biting**	0	−**
**righting reflex**	−*	−***
**contact righting reflex**	0	0
**negative geotaxis**	0	0
**aggression**	0	−*
**body temperature**	0	0
**body weight**	−***	−**

− = decrease, + = increase, 0 = no change, nm = not measured.

*p<0.05,

**p<0.01,

***p<0.001 for the significance of the difference between Pv-Δγ2 or Pv-Δγ2-partial rescue and control γ2I77 mice (Mann-Whitney U-test, Wilcoxon signed rank test or Student's *t*-test). n = 15–22 per line.

The adult male Pv-Δγ2 mice were small (20.6±3 g vs. 25.9±3 g for γ2I77 mice, Student's *t*-test p<0.001), dragged their tail and had a clearly visible walking deficit (flattened pelvic elevation, low belly, wide step, hind legs wide apart), but they nevertheless moved normally and actively in home cages. They also had light, intermittent tremor and abnormal trunk curl when lifted by the tail. Their increase in body weight during 6 months (from 2 to 8 months of age) was smaller than in γ2I77 control mice, and their food intake per day was also smaller (p<0.05) at the age of 6 months. The quality of body fur was abnormal (greasy, matted) compared with controls, possibly due to deficient grooming. The phenotype of Pv-Δγ2-partial rescue mice was similar in many respects to that of Pv-Δγ2 mice (Table 1), but their gait, pelvic elevation and fur quality were normal unlike in Pv-Δγ2 mice. Despite a consistently lower body weight (19.8±1 g vs. 23.3±3 g for γ2I77 mice, p<0.01) and a slower increase in body weight from 2 to 8 months of age compared with controls (p<0.05), their food intake at 6 months was similar to control mice.

Specific behavioral investigations of the Pv-Δγ2 and Pv-Δγ2-partial rescue mice are given below.

### Decreased anxiety-like behavior in Pv-Δγ2 and Pv-Δγ2-partial rescue mice

**Figure 4:**
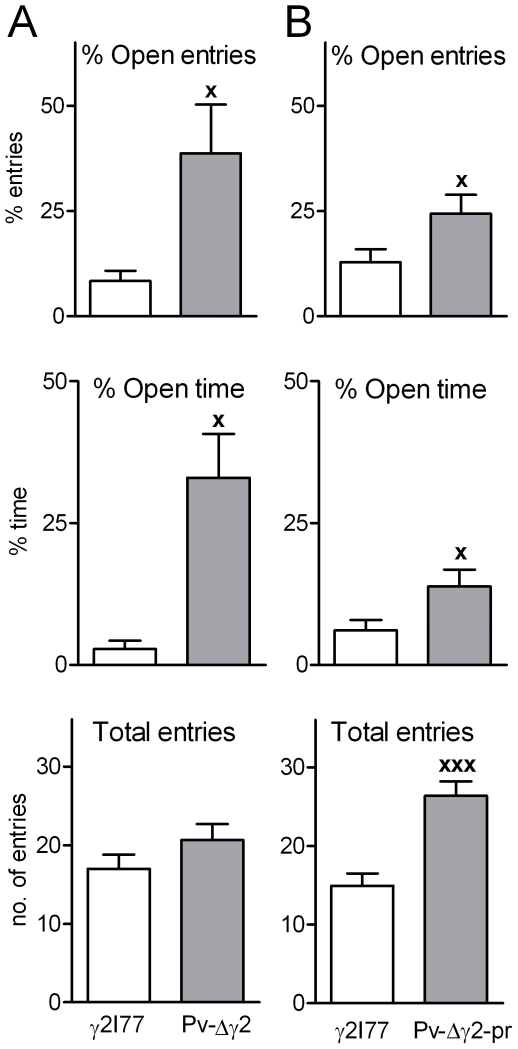
Comparison of the mouse lines for anxiety-related behaviors in elevated plus-maze test. (**A**) Basal anxiety-related behaviors of Pv-Δγ2 mice (n = 7) and control littermate γ2I77 mice (n = 8) in the elevated plus-maze test. (**B**) Basal anxiety-related behavior level of Pv-Δγ2-partial rescue mice (n = 11), wild type γ2F77 (n = 5) and control littermate γ2I77 mice (n = 6). Data from wild-type and γ2I77 mice were pooled, since there were no significant differences between them. ^x^ p<0.05, ^xxx^ p<0.001 for the difference between the mouse lines (Student's *t*-test). Data are presented as means ± SEM.

Pv-Δγ2 (n = 7) and γ2I77 (n = 8) mice and Pv-Δγ2-partial rescue (n = 11) with wild-type γ2F77 (n = 5, littermates of γ2I77 mice) and γ2I77 (n = 6) control mice were tested in the elevated plus-maze apparatus to evaluate their basal level of anxiety. Pv-Δγ2 mice entered the open arms of the maze more frequently (p<0.05, Student's *t*-test) and spent more time in the open arms (p<0.05) than control mice (Figure 4A). Pv-Δγ2-partial rescue mice also entered the open arms of the maze more frequently (p<0.05) and spent more time in the open arms (p<0.05) than control mice (Figure 4B). However, the overall motor activity of Pv-Δγ2-partial rescue mice was higher than that of the control mice (p<0.001; Figure 4B).

### Increased acoustic startle in Pv-Δγ2-partial rescue mice; decreased prepulse inhibition in both Pv-Δγ2 and Pv-Δγ2-partial rescue mice

**Figure 5:**
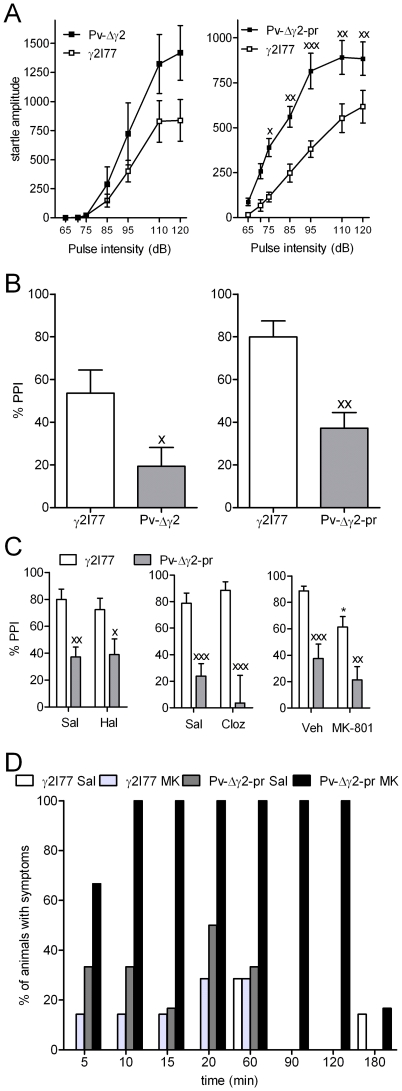
Assessment of sensorimotor coordination in Pv-mouse lines using tests for startle response and prepulse inhibition of startle. (**A**) Acoustic startle reflex reaction curves of Pv-Δγ2 and Pv-Δγ2-partial rescue mice. Pulse intensity was raised from 65 to 120 dB. Data for Pv-Δγ2 (n = 7), Pv-Δγ2-partial rescue (n = 15) and control littermate γ2I77 mice (n = 8 for Pv-Δγ2 mice and n = 15 for Pv-Δγ2-partial rescue mice) are presented as means ± SEM. ^x^ p<0.05, ^xx^ p<0.01, ^xxx^ p<0.001 for the difference between Pv-Δγ2-partial rescue and control γ2I77 lines (repeated measures ANOVA and Newman-Keuls *post hoc* test). (**B**) Prepulse inhibition (PPI) of the acoustic startle reflex in Pv-Δγ2 and Pv-Δγ2-partial rescue mice. Data for Pv-Δγ2 (n = 7), Pv-Δγ2-partial rescue (n = 15) and control littermate γ2I77 mice (n = 8 for Pv-Δγ2 mice and n = 15 for Pv-Δγ2-partial rescue mice) are presented as means ± SEM. ^x^ p<0.05, ^xx^ p<0.01 for the difference between Pv-Δγ2 or Pv-Δγ2-partial rescue line and γ2I77 line (Student's *t*-test). (**C**) The effects of 0.5 mg/kg haloperidol, 4 mg/kg clozapine and 0.15 mg/kg MK-801 on the PPI of Pv-Δγ2-partial rescue mice. Data for Pv-Δγ2-partial rescue (n = 12) and control littermate γ2I77 mice (n = 15) are presented as means ± SEM. ^x^ p<0.05, ^xx^ p<0.01, ^xxx^ p<0.001 for the difference between the mouse lines (two-way ANOVA and Newman-Keuls *post hoc* test). * p<0.05 for the difference between the drug and vehicle in γ2I77 mice. (**D**) Average symptom score of agitation syndrome in Pv-Δγ2-partial rescue (n = 12) and control littermate γ2I77 mice (n = 15) during 3 h after 0.15 mg/kg MK-801 administration. Mice were scored visually for the presence or absence of jumping, hyperlocomotion, stereotypic behavior and seizures.

The increase in acoustic startle reflex of Pv-Δγ2 and Pv-Δγ2-partial rescue mice noted in the SHIRPA screen (Table 1) was investigated further in acoustic startle chambers. Pv-Δγ2 (n = 7) mice had a trend towards increased amplitudes of startle reflex (Figure 5A) and the Pv-Δγ2-partial rescue mice (n = 12) had significantly increased startle reflex at sound stimulus intensities from 75 to 120 dB compared with control γ2I77 mice (n = 15) (sound intensity×line interaction F_1,174_ = 2.72, p<0.05, repeated measures two-way ANOVA; Figure 5A). Different values for control γ2I77 mice in these two separate tests are due to adjustments in detection sensitivity.

PPI of the acoustic startle reflex of both Pv-Δγ2 mice (p<0.05, Student's *t*-test) and Pv-Δγ2-partial rescue mice (p<0.01) was decreased compared with that of γ2I77 mice (Figure 5B). Acute “antipsychotic treatment” (haloperidol 0.5 mg/kg or clozapine 4 mg/kg) did not restore the PPI of Pv-Δγ2-partial rescue mice to a normal level (Figure 5C; not studied in Pv-Δγ2 mice), nor did acute administration of a psychotomimetic N-methyl-D-aspartate receptor antagonist MK-801 (0.15 mg/kg) further deteriorate it, though MK-801 reduced %PPI in γ2I77 control mice (Figure 5C).

In Pv-Δγ2-partial rescue mice MK-801 (0.15 mg/kg) produced an agitation syndrome lasting for over 120 min but produced no overt symptoms in control γ2I77 mice. Pv-Δγ2-partial rescue and γ2I77 mice were scored visually for the presence or absence of agitation symptoms (jumping, hyperlocomotion, stereotypic behavior, seizures) for 3 h after the MK-801 injection in home cages (Figure 5D).

### Impaired spatial learning in Pv-Δγ2 mice

**Figure 6:**
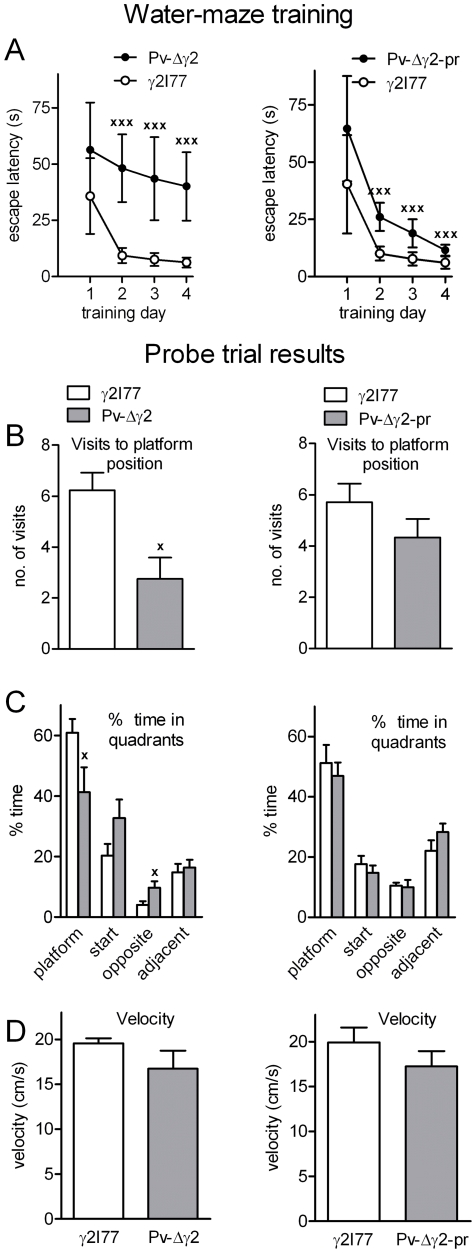
Spatial learning and memory in Pv-Δγ2 mouse models. (**A**) Morris water maze training of Pv-Δγ2, Pv-Δγ2-partial rescue and γ2I77 mice. Mice were trained during four days to find a submerged platform in a pool of water. Latency to reach the platform was recorded. Data are presented as daily averages ± SEM. ^xxx^ p<0.001 for the difference between Pv and γ2I77 mice (one-way ANOVA and Newman-Keuls *post hoc* test). (**B**) The number of visits to the former platform position during the probe trial. ^x^ p<0.05 for the difference between Pv-Δγ2 and γ2I77 mouse lines, Student's *t*-test. (**C**) Time spent in the four quadrants during the probe trial. ^x^ p<0.05 for the difference between Pv-Δγ2 and γ2I77 mouse lines, Student's t-test. (**D**) Swimming velocity of Pv-Δγ2, Pv-Δγ2-partial rescue and γ2I77 mice in the probe trial.

Pv-Δγ2, Pv-Δγ2-partial rescue and γ2I77 control mice were trained during 4 days to find a submerged platform in a pool of water. The spatial learning of both Pv mouse lines was impaired as can be seen from longer latencies to find the platform during training (Figure 6A), but the learning of Pv-Δγ2-partial rescue mice appears less severely compromised than that of Pv-Δγ2 mice.

During the probe trial, the platform was removed and visits by the mouse to the former platform quadrant as well as the time spent in different quadrants were recorded. Pv-Δγ2 mice had fewer platform quadrant visits (p<0.05, Student's *t*-test) (Figure 6B) and spent less time spent in the platform quadrant, compared to their γ2I77 control mice (p<0.05) (Figure 6C). They also spent more time in the quadrant opposite the platform quadrant (p<0.05). Pv-Δγ2-partial rescue mice had a similar number of platform quadrant visits (Figure 6B) and spent a similar amount of time in the platform quadrant as their γ2I77 control mice (Figure 6C). The swimming velocities during the whole one-min probe trial were similar in all mouse lines (Figure 6D), so the longer latencies by the Pv-lines to find the platform are more likely due to learning/memory deficits than to motor disturbances.

### Impaired motor performance and learning

**Figure 7:**
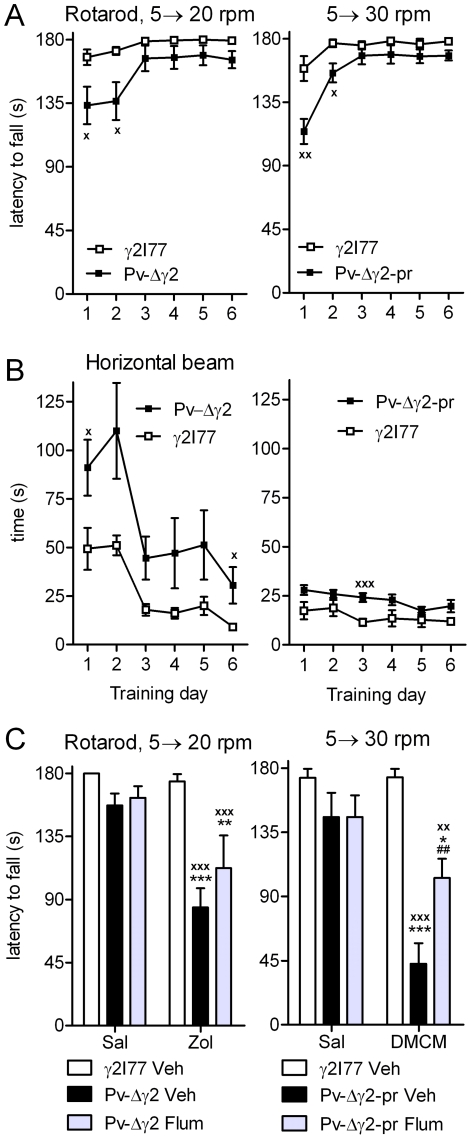
Motor performance and motor pharmacological sensitivity of Pv-Δγ2, Pv-Δγ2-partial rescue and control littermate γ2I77 mice. (**A**) Motor training of Pv-Δγ2 (n = 7), Pv-Δγ2-partial rescue (n = 9) and control littermate γ2I77 mice (n = 8 for Pv-Δγ2 mice and n = 9 for Pv-Δγ2-partial rescue mice). Mice were trained during 6 days to stay for 180 s on a rotating rod accelerated from 5 to 20 rpm for Pv-Δγ2 mice and from 5 to 30 rpm for Pv-Δγ2-partial rescue mice. Latency to fall from the rod was recorded. (**B**) The mice were also trained to traverse a 1-m wooden beam (diameter 0.8 cm). Time to traverse the beam was recorded. Data are presented as daily averages ± SEM. ^x^ p<0.05, ^xx^ p<0.01, ^xxx^ p<0.001 for the difference in the corresponding latencies or times to traverse between the mouse lines. (**C**) The effect of 20 mg/kg zolpidem in Pv-Δγ2 mice on the rotarod; pretreatment with 15 mg/kg flumazenil. The effect of 3 mg/kg DMCM in Pv-Δγ2-partial rescue mice; pretreatment with 15 mg/kg flumazenil. ^xx^ p<0.01, ^xxx^ p<0.001 for the difference between the mouse lines; * p<0.05, ** p<0.01, *** p<0.001 for the difference between zolpidem and saline or DMCM and saline; ^##^ p<0.01 for the difference between vehicle and flumazenil in DMCM-treated mice (repeated measures ANOVA and Newman-Keuls *post hoc* test).

To investigate the motor capability of Pv-Δγ2 (n = 7) and Pv-Δγ2-partial rescue (n = 9) mice, their rotarod performance was measured. Pv-Δγ2 mice learned the rotarod task more slowly than γ2I77 (n = 8) control mice, as demonstrated by shorter latencies to fall from the rod, even in an easy, 5 to 20 rpm acceleration task (Figure 7A). There was a genotype difference between Pv-Δγ2 and γ2I77 control mice during training (F_1,89_ = 5.39, p<0.05, repeated measures two-way ANOVA), being significant on days 1 and 2 (p<0.05, Student's *t*-test). The motor learning of Pv-Δγ2 mice was also impaired on the walking beam during the 6-day training period (genotype effect: F_1,89_ = 6.32, p<0.05; Figure 7B). There was a significant difference between lines on days 1 and 6 (p<0.05).

Generally, the Pv-Δγ2-partial rescue mice had less impairment than the Pv-Δγ2 mice, and they could be trained to perform a more difficult task with a rotating rod speed accelerating from 5 to 30 rpm. There was nevertheless a genotype effect (F_1,95_ = 8.95, p<0.001, repeated measures two-way ANOVA; Figure 7A), which was significant on day 1 (p<0.01, Student's *t*-test) and day 2 (p<0.05). The performance of Pv-Δγ2-partial rescue mice on the walking beam was almost normal (Figure 7B), but there was still a genotype effect (F_1,95_ = 6.89, p<0.05), with the beam traversing time being significantly longer on day 3 when compared with γ2I77 mice (n = 9; p<0.001).

### Altered sensitivity of motor performance to zolpidem and DMCM

After establishing a stable level of rotarod performance, pharmacological sensitivities of Pv-Δγ2 and Pv-Δγ2-partial rescue mice to GABA_A_ receptor modulators were investigated. The mice were administered zolpidem (cumulative 20+20 mg/kg) or DMCM (3 mg/kg) and tested 1–15 min later for the latency to fall down from the rotarod.

The motor-impairing effects of 20+20 mg/kg zolpidem were increased in Pv-Δγ2 mice (genotype effect: F_1,42_ = 9.610, p<0.001, repeated measures two-way ANOVA, data not shown). The effects of a single 20 mg/kg dose of zolpidem in Pv-Δγ2 mice could not be reversed by pretreatment with 15 mg/kg flumazenil (Figure 7C). The falling latency of Pv-Δγ2-partial rescue mice did not differ from control mice after zolpidem (data not shown).

DMCM (3 mg/kg) had a motor-impairing effect in Pv-Δγ2-partial rescue mice (Figure 7C; F_1,30_ = 14.62, p<0.001), but not in Pv-Δγ2 mice (data not shown) nor in γ2I77 control mice. Flumazenil pretreatment (15 mg/kg) significantly reversed the effect of DMCM in Pv-Δγ2-partial rescue mice (Figure 7C). Importantly, DMCM (3 mg/kg) produced a marked change in the body posture, decreasing pelvic elevation and flattening the body and tail onto the floor, without producing apparent sedation.

### Unusual harmaline-induced tremor in Pv-Δγ2 and Pv-Δγ2-partial rescue mice

The Pv-Δγ2 (n = 7) and Pv-Δγ2-partial rescue (n = 9) mice had a mild visible tremor in SHIRPA screening (Table 1). These mice and γ2I77 control mice (n = 7+8) were further tested with a piezoelectric weight sensor. However, the tremor at baseline conditions was too slight to be detected with the apparatus, and therefore, we provoked tremor with the β-carboline alkaloid harmaline (e.g. [60], [61]). When administered 15 mg/kg harmaline, γ2I77 mice developed a constant tremor of 12 Hz, with a mean amplitude of 5.8±1.5 tremor units. Although Pv-Δγ2 and Pv-Δγ2-partial rescue mice also developed harmaline-tremor, it was variable in frequency (8–28 Hz) and episodic (14±6 episodes during 300 s in Pv-Δγ2 mice; 16±7 episodes in Pv-Δγ2-partial rescue mice). The amplitude at the dominant frequency was 1.8±0.6 tremor units in Pv-Δγ2 mice and 3.1±2.5 tremor units in Pv-Δγ2-partial rescue mice.

### Decreased thermal pain reactivity and increased analgesic effect of DMCM

**Figure 8:**
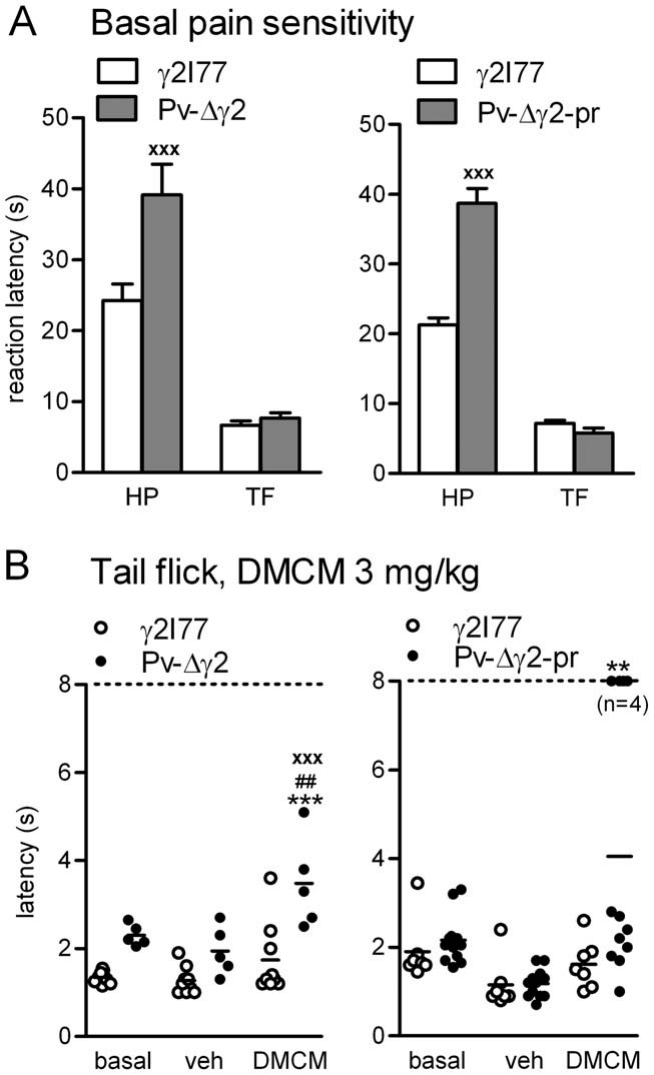
Nociceptive responses of the Pv-Δγ2 mouse models. (**A**) Basal thermal pain reactivity of Pv-Δγ2 and Pv-Δγ2-partial rescue mice in hot plate (HP) and tail flick (TF) tests. Data for Pv-Δγ2 (n = 5), Pv-Δγ2-partial rescue (n = 11) and control littermate γ2I77 mice (n = 9 for Pv-Δγ2 mice and n = 8 for Pv-Δγ2-partial rescue mice) are presented as means ± SEM. ^###^ p<0.001 for the difference between the mouse lines (Student's *t*-test). (**B**) The effect of 3 mg/kg DMCM on thermal pain in the tail flick test. ^##^ p<0.01 for the difference compared with basal value (two-way repeated measures ANOVA and Newman-Keuls *post hoc* test). ** p<0.01, *** p<0.001 for the difference compared with vehicle injection. ^xxx^ p<0.001 for the difference between the mouse lines.

Both Pv-Δγ2 (n = 5) and Pv-Δγ2-partial rescue (n = 11) mice had a significantly (p<0.001, Student's *t*-test) increased reaction latency to hot plate-induced thermal pain sensation compared with control γ2I77 mice (n = 9+8; Figure 8A).

In the tail flick test, both Pv-lines had similar latencies as the control mice (Figure 8A), indicating that their spinal pain reflexes were normal. However, when the Pv-Δγ2 and Pv-Δγ2-partial rescue mice were tested for tail flick responses after DMCM (3 mg/kg) we found evidence for an analgesic effect (Figure 8B). In Pv-Δγ2 mice, DMCM slightly lengthened the latency for tail flick compared with γ2I77 control mice (p<0.05, two-way ANOVA and Newman-Keuls *post hoc*-test), also when compared with the vehicle values (p<0.01). In the experiment with Pv-Δγ2-partial rescue mice, there was a hyperalgesic effect after vehicle treatment in all animals. DMCM produced a full analgesic response (no tail flick until the 8 s cut-off) in a subgroup of Pv-Δγ2-partial rescue mice, while two-thirds of them were insensitive to it. Using another group (n = 7) of Pv-Δγ2-partial rescue mice, we confirmed that about one half (n = 4) of the animals did not react to a noxious thermal stimulus within the cut-off time after receiving DMCM (6 mg/kg), while the insensitive mice had unchanged latencies (data not shown).

## Discussion

We studied mice in which fast synaptic inhibition onto Pv-neurons has been abolished [6]. Pv-Δγ2 and Pv-Δγ2-partial rescue mice had alterations in many behavioral modalities, including a decreased level of anxiety-like behavior, impaired motor coordination, altered expression of tremor, a heightened acoustic startle reflex and decreased prepulse inhibition of acoustic startle, in addition to changes in pharmacological sensitivity to GABAergic substances and the N-methyl-D-aspartate receptor antagonist MK-801. We could also map alterations of GABA_A_ receptor properties in a number of brain regions (especially subcortical, brainstem and spinal regions) of the Pv-Δγ2 and Pv-Δγ2-partial rescue mice. Given the wide distribution of Pv cells in the CNS, such a range of behavioral phenotypes was perhaps expected, even though the disruption was not lethal.

There were several physical alterations that allowed both Pv-Δγ2 and Pv-Δγ2-partial rescue mice to be visually identified, but the most prominent features were reduced food intake and low body weight. Decreases in GABAergic tone in hypothalamic regions important for the control of feeding [62] lead to anorexia and starvation in mice [63]; and conversely GABAergic agonists, such as benzodiazepines, promote feeding [64]. Our findings suggest that on a population of hypothalamic Pv-neurons, the lack of fast synaptic inhibition may, directly or indirectly, produce the reduced food intake and lower body weight characteristic of Pv-Δγ2 and Pv-Δγ2-partial rescue mice.

### Motor deficits

The motor deficits of Pv-Δγ2 mice could be rescued to some extent by expression of the γ2 subunit in a subset of Pv-cells (Purkinje/stellate/basket cells in the cerebellum and neurons of the reticular thalamic nucleus; Figures 1 and 2). The haplo-insufficiency phenotypes found with heterozygote γ2 total knockout mice [27], [29] indicates that there is little, if any, spare γ2 subunit capacity; because the γ2 re-expression in the cerebellum and reticular thalamus of Pv-Δγ2-partial rescue mice was still substantially below wild-type levels (as measured by [^3^H]Ro 154513 binding, Figure 2), this probably accounts for some of the residual motor phenotype. Furthermore, Pv-positive interneurons at the level of spinal cord neuronal networks are involved in coordinating motor patterns [65], [66], and the spinal cord had deficient GABA_A_ receptors in both Pv-Δγ2 and Pv-Δγ2-partial rescue mice (Figure 3C). Reduced fast inhibition onto these interneurons may have directly impaired motor function of the Pv mouse lines.

Surprisingly, the motor deficits in Pv-Δγ2 mice were also aggravated by administration of a high dose of the benzodiazepine agonist zolpidem. As the background γ2I77 line is globally insensitive to conventional zolpidem doses [11], [33], [34], and Pv-Δγ2 mice are missing the γ2 subunit from Pv-neurons, another mechanism is needed to explain this sensitivity. We have previously discovered that the γ2I77 line is sensitive to high (40–60 mg/kg) zolpidem doses [55], possibly via inhibition of neuronal Ca^2+^ channels [67], [68]. No zolpidem effect was observed in control γ2I77 mice in the present study, probably because the 5 to 20 rpm task was excessively easy for them to perform. It is also important to note that the impairment caused by zolpidem in Pv-Δγ2 mice could not be reversed by flumazenil (Ro 15-1788) pretreatment, which points to an action through a non-αβγ2 binding site or a non-GABA_A_ receptor mechanism.

Compared with Pv-Δγ2 mice, it is likely that in Pv-Δγ2-partial rescue mice the basal activity of Pv-neurons is partially normalized due to the presence of fast synaptic inhibition on the reticular thalamic neurons and some cerebellar cells. This could explain the differences in pharmacological sensitivity of motor performance observed between Pv-Δγ2 and Pv-Δγ2-partial rescue mice. Whereas DMCM had no motor-impairing effect in Pv-Δγ2 mice, it caused a robust decrease in the latency to fall from the rotarod in Pv-Δγ2-partial rescue mice, which could be significantly reversed by the benzodiazepine antagonist flumazenil pretreatment, thus showing that the effect was due to αβγ2-type receptors. In wild-type αβγ2F77 receptors, DMCM is an inverse agonist, decreasing the effects of GABA via the benzodiazepine binding site. We hypothezise that DMCM decreases the effects of the inhibitory inputs onto γ2F77^GFP^-expressing Pv cells and thus increases the inhibitory load towards the non-Pv cells which contain insensitive γ2I77 GABA_A_ receptors leading to motor impairment. The lack of zolpidem effect in Pv-Δγ2-partial rescue mice was unexpected, since the presence of wild-type γ2F77 subunits in the cerebellum should lead to some rescue of zolpidem as well as DMCM sensitivity (cf. [11]). However, in the cerebellar Purkinje cell γ2F77 rescue mice different pharmacology of motor performance was observed as zolpidem impaired the rotarod performance [11]. This indicates that the cerebellar cortical output is delicately balanced and can be differentially modulated by systemic activators and inhibitors.

Based on the basal behavioral dissimilarities between Pv-Δγ2 and Pv-Δγ2-partial rescue mice, γ2 restoration in Pv cells in additional brain areas may also have happened, but the amount of restored γ2F77 protein may be too small to be detected by ligand autoradiography (e.g. there is a faint transgene signal detected by *in situ* hybridization in the colliculi, Figure 1C).

### Altered tremor induction, reduced pain sensitivity and sensorimotor impairment

The readout from behavioral and pharmacological experiments with living mice is often from motor responses/activity, even though a given test purports to measure e.g. anxiety or memory. Therefore, the results from other tests have to be interpreted in the light of baseline differences in motor functions.

There was slight tremor in both mouse model lines in the observational test (Table 1), and therefore, we wanted to test whether this feature would be exaggerated by the well-known tremorogenic β-carboline compound harmaline [69]. The molecular mechanism of action of harmaline is poorly known, but our results indicate that it does not lose its efficacy in γ2I77 mice that lose the high-affinity binding for the β-carboline DMCM. Interestingly, the harmaline-induced tremor in both Pv-Δγ2 and Pv-Δγ2-partial rescue mice was altered from rather constant high-amplitude tremor to more episodic low-amplitude activity. Thus, it is possible that the Pv-cells in the well-known tremor pathway from the inferior olivary nucleus to the cerebellar cortex are poorly functioning in the mutants [70].

GABA_A_ receptor α2/3 subunit-selective agonists have shown promise as analgesics, highlighting the antinociceptive possibilities of positive modulation of the receptor [71]. Both Pv-Δγ2 and Pv-Δγ2-partial rescue mice had impaired pain sensitivity in the hot-plate test that is usually interpreted to measure central rather than spinal pain pathways [72]. The mouse lines demonstrated unaltered latencies to thermal stimuli of the tail, which elicits a quick reflexive flick. Pv-cells are present from the spinal cord to cerebral cortex, giving many opportunities for modulation of pain perception in Pv-Δγ2 and Pv-Δγ2-partial rescue mice, thus making the localization(s) of the modulation difficult. In any case, GABAergic neurons in the spinal lamina II contact pain-conducting myelinated AΔ and unmyelinated C-fibers [73], and pharmacological blockade of GABA_A_ receptors produces tactile allodynia [74]. If the overall GABAergic tone of the spinal cord is increased in Pv-Δγ2 mice due to increased activity of Pv-neurons, pain sensitivity might consequently decrease. DMCM produced a slight analgesic reaction in Pv-Δγ2 mice, unlike in control γ2I77 mice. As DMCM cannot exert its inverse agonistic effects prevalent in wild-type γ2F77 animals, it may have acted as a positive modulator via the loreclezole site, where DMCM has a clear agonistic action [33], [75]. In the Pv-Δγ2-partial rescue mice the situation was different, as DMCM either produced a full analgesic effect or failed to alter the latency of the tail flick at all (Figure 8). Since DMCM induced a loss of muscle tone without any reduction of spontaneous locomotor activity, a subset of the Pv-Δγ2-partial rescue mice might have been unable to produce the normal nociceptive reflex. It is also possible that their sensory perception had failed to register the pain when under the influence of DMCM. As an alternative possibility, the cerebellum of Pv-Δγ2-partial rescue mice with its wild-type γ2F77 subunit-containing GABA_A_ receptors, might mediate this abnormal DMCM-induced antinociception.

The decreased PPI of acoustic startle we found in Pv-Δγ2 and Pv-Δγ2-partial rescue mice points to a disruption of network function at the inferior colliculus or thalamocortical level. It might reflect a dysfunction of sensorimotor integration [76], [77]. Impaired PPI is present both in human schizophrenia patients [78], [79] and animal models of schizophrenia (for a review, see e.g. [80]), but we failed to see any clear reduction of the impairment of PPI by the antipsychotic drugs clozapine and haloperidol (Figure 5C). In respect to defective PPI in Pv-Δγ2 mice, it was notable that Pv-Δγ2-partial rescue mice were more sensitive to other behavioral effects of the N-methyl-D-aspartate receptor antagonist MK-801, although it could not enhance their defects in PPI (Figure 5D). The mechanisms of this interesting pharmacological effect are unknown. There is ample evidence for the potency of GABA_A_ agonistic benzodiazepines to attenuate the hyperactivity induced by MK-801 and phencyclidine (another N-methyl-D-aspartate receptor antagonist) [81], [82] and for the cortical Pv-interneurons being an important target for MK-801 and phencyclidine actions [83], [84]. Thus, we suggest that abnormal Pv-neuron activity in Pv-Δγ2 mice has made the animals more vulnerable to toxic effects by N-methyl-D-aspartate receptor antagonists.

### Emotional and cognitive deficits: reduced anxiety and impaired spatial memory

Pv cells that express GABA_A_ receptors are found in many amygdaloid nuclei [2], [85], and also in the ventral hippocampus, another brain region which contributes to emotional behavior [86]. Indeed, knockout mice for the GABA synthesizing enzyme glutamic acid decarboxylase 65 have increased anxiety [87]. Similarly, mice heterozygous for knockout of the γ2 subunit gene, or mice with a 65% knockdown of global γ2 gene expression, have increased anxiety levels [27], [28], in agreement with less GABAergic tone in the amygdala and other circuits. A possible increase in the activity of Pv-interneurons in Pv-Δγ2 and Pv-Δγ2-partial rescue mice would increase the overall GABAergic tone in the amygdala, presumably leading to mice with less anxiety. This response might correlate with the altered effects of 2 µM and 1 mM GABA on the anion channel binding site labeled by [^35^S]TBPS in the central nucleus of amygdala of Pv-Δγ2 and Pv-Δγ2-partial rescue mice, respectively (Figure 3B, Table S1).

Because of the strongly altered hippocampal oscillations in the Pv-Δγ2 mice [6], we predicted that the mice would show impaired working memory; but our experiments using the spontaneous alternation task showed no differences of the scores for Pv-Δγ2 and Pv-Δγ2-partial rescue mice as compared with control γ2I77 mice (data not shown). These results were inconclusive as none of the mouse lines performed well in this task (only about 50% alternation), in contrast with several other control mouse lines we have observed to perform consistently better [88]. On the other hand, it took longer for both Pv-Δγ2 and Pv-Δγ2-partial rescue mice to find the escape platform in the Morris water maze task, and the latencies of the Pv-Δγ2 mice were still much longer after 4 days of training. This poor learning could be seen as shorter times spent in the platform quadrant in the probe trials. Hypothermia, especially in frail gene-modified mice, might have impaired learning in the water maze [89]; on the other hand, this is not likely since the mice were quickly warmed up between trials. Although in the probe trial the swimming speed of the mutant mice did not differ from those of the controls, as discussed above a part of the impaired learning could actually originate from deficits in motor performance.

### Conclusion

Given the huge diversity of Pv neurons and the neuronal circuits in which they participate, it would be unrealistic to relate the behavioral defects seen in the Pv-Δγ2 and Pv-Δγ2-partial rescue mice to any one particular human neurological or psychiatric disease. However, it is clear that γ2 subunit-containing GABA_A_ receptors in Pv-positive cells, and by implication, fast synaptic inhibition onto these cells is essential for normal brain function. The processes we found defective are quite disparate: from uncoupling of cortical network oscillations [6], reduced anxiety through to impaired spatial memory to reduced pain sensitivity. For the interpretation of behavioral experiments, this makes it challenging to untangle cause and effect. Nevertheless, some of these components could be separately investigated, perhaps using knockout of γ2 gene expression selectively in Pv-cells in particular brain regions, such as we have already done for cerebellar Purkinje cells [11], [12].

## Supporting Information

Table S1 **Binding of [^35^S]TBPS in various brain areas of γ2I77 and Pv-Δγ2 and Pv-Δγ2-partial rescue (pr) mice in the absence and presence of 2 µM and 1 mM GABA.**
(DOC)
