# Supplementary material for: Removal of GABAA Receptor γ2 Subunits from Parvalbumin Neurons Causes Wide-Ranging Behavioral Alterations
Source: PLoS One. 2011 Sep 2;6(9):e24159. doi: 10.1371/journal.pone.0024159 (PMC3166293; doi:10.1371/journal.pone.0024159)
Supplement: Table S1 — Binding of [35S]TBPS in various brain areas of γ2I77 and Pv-Δγ2 and Pv-Δγ2-partial rescue (pr) mice in the absence and presence of 2 µM and 1 mM GABA. (DOC) [file pone.0024159.s001.doc]

**Removal of GABA_A_ Receptor γ2 Subunits from Parvalbumin Neurons Causes Wide-ranging Behavioral Alterations**

Supporting information

**Table S1**

Binding of [^35^S]TBPS in various brain areas of γ2I77 and Pv-Δγ2 and Pv-Δγ2-partial rescue (pr) mice in the absence and presence of 2 μM and 1 mM GABA.

| **Brain region** | **Mouse line** | **Basal binding**  **(nCi/g)** | **GABA 2 μM**  **(% of basal)** | **GABA 1 mM**  **(% of basal)** |
| --- | --- | --- | --- | --- |
| *Cerebral cortex* | γ2I77 | 176±40 | 24±6 | 2±1 |
|  | Pv-Δγ2 | 163±51 | 25±5 | 3±1 |
|  | Pv-Δγ2-pr | 148±42 | 26±6 | 7±3*^,♦^ |
| *Olfactory bulb* | γ2I77 | 37±7 | 19±3 | 1±2 |
|  | Pv-Δγ2 | 31±3 | 12±2 | 1±1 |
|  | Pv-Δγ2-pr | 31±18 | 22±14 | 6±5 |
| *Lateral septum* | γ2I77 | 188±15 | 34±5 | 4±2 |
|  | Pv-Δγ2 | 166±27 | 39±9 | 3±0 |
|  | Pv-Δγ2-pr | 157±52 | 28±3 | 8±3**^,♦♦^ |
| Basal ganglia |  |  |  |  |
| *Nucleus accumbens* | γ2I77 | 62±19 | 38±9 | 2±1 |
|  | Pv-Δγ2 | 74±14 | 25±2* | 1±1 |
|  | Pv-Δγ2-pr | 56±14 | 36±8 | 6±3**^,♦^ |
| *Caudate-putamen* | γ2I77 | 59±4 | 62±9 | 4±3 |
|  | Pv-Δγ2 | 59±10 | 60±6 | 2±1 |
|  | Pv-Δγ2-pr | 61±6 | 60±11 | 7±3^♦^ |
| *Globus pallidus* | γ2I77 | 219±30 | 83±13 | 3±2 |
|  | Pv-Δγ2 | 213±14 | 82±4 | 3±1 |
|  | Pv-Δγ2-pr | 228±19 | 48±10***^,♦♦♦^ | 7±3**^,♦^ |
| Hypothalamus |  |  |  |  |
| *Medial preoptic area* | γ2I77 | 67±26 | 69±20 | 3±2 |
|  | Pv-Δγ2 | 88±37 | 56±11 | 2±1 |
|  | Pv-Δγ2-pr | 64±15 | 58±18 | 12±7**^,♦♦^ |
| *Paraventricular nucleus* | γ2I77 | 81±44 | 97±59 | 2±2 |
|  | Pv-Δγ2 | 67±20 | 69±37 | 4±3 |
|  | Pv-Δγ2-pr | 73±19 | 59±37 | 12±9 |
| Amygdala, hippocampus |  |  |  |  |
| *Basolateral amygdala* | γ2I77 | 87±23 | 19±3 | 2±1 |
|  | Pv-Δγ2 | 59±12 | 18±5 | 2±1 |
|  | Pv-Δγ2-pr | 90±32 | 17±4 | 5±1**^,♦♦^ |
| *Med. div.of central amygdala* | γ2I77 | 62±16 | 33±8 | 2±2 |
|  | Pv-Δγ2 | 49±3 | 62±15** | 1±0 |
|  | Pv-Δγ2-pr | 60±14 | 33±12^♦♦^ | 6±3*^,♦^ |
| *Hippocampal CA1* | γ2I77 | 91±18 | 27±8 | 5±2 |
|  | Pv-Δγ2 | 87±22 | 26±6 | 4±2 |
|  | Pv-Δγ2-pr | 100±38 | 25±7 | 7±3 |
| *Hippocampal CA3* | γ2I77 | 68±19 | 42±14 | 3±2 |
|  | Pv-Δγ2 | 58±17 | 35±7 | 4±2 |
|  | Pv-Δγ2-pr | 77±22 | 30±10 | 6±3 |
| *Dentate gyrus* | γ2I77 | 84±35 | 28±15 | 4±3 |
|  | Pv-Δγ2 | 57±10 | 41±29 | 2±2 |
|  | Pv-Δγ2-pr | 78±21 | 31±17 | 5±2 |
| *Thalamus* | γ2I77 | 114±15 | 50±7 | 11±5 |
|  | Pv-Δγ2 | 113±22 | 48±7 | 11±2 |
|  | Pv-Δγ2-pr | 116±27 | 39±5** | 15±3 |
| *Thalamic med. geniculate n.* | γ2I77 | 173±50 | 53±17 | 7±2 |
|  | Pv-Δγ2 | 153±16 | 44±6 | 7±1 |
|  | Pv-Δγ2-pr | 166±37 | 40±8 | 12±4*^,♦^ |
| Midbrain, pons, spinal cord |  |  |  |  |
| *Substantia nigra p.reticulata* | γ2I77 | 222±34 | 37±7 | 3±2 |
|  | Pv-Δγ2 | 221±25 | 28±5* | 8±2** |
|  | Pv-Δγ2-pr | 231±23 | 22±3** | 8±4* |
| *Ventral tegmental area* | γ2I77 | 80±24 | 93±31 | 2±2 |
|  | Pv-Δγ2 | 159±74* | 25±2*** | 3±2 |
|  | Pv-Δγ2-pr | 149±44* | 30±6*** | 7±5*^,♦^ |
| *Periaqueductal gray area* | γ2I77 | 141±25 | 63±16 | 2±2 |
|  | Pv-Δγ2 | 164±13 | 35±7** | 7±2*** |
|  | Pv-Δγ2-pr | 208±17*** | 26±3*** | 11±3***^,♦^ |
| *Superior colliculus* | γ2I77 | 202±45 | 46±12 | 2±2 |
|  | Pv-Δγ2 | 206±50 | 40±12 | 5±1 |
|  | Pv-Δγ2-pr | 209±37 | 28±6 | 8±4** |
| *Inferior colliculus* | γ2I77 | 192±50 | 43±9 | 4±3 |
|  | Pv-Δγ2 | 221±36 | 31±6 | 8±2* |
|  | Pv-Δγ2-pr | 225±28 | 26±3 | 9±4* |
| *Raphe nucleus* | γ2I77 | 110±17 | 75±17 | 1±2 |
|  | Pv-Δγ2 | 169±28** | 37±10*** | 7±3* |
|  | Pv-Δγ2-pr | 158±30** | 30±3*** | 9±6** |
| *Pons* | γ2I77 | 38±5 | 104±18 | 0±0 |
|  | Pv-Δγ2 | 43±15 | 70±20** | 6±2** |
|  | Pv-Δγ2-pr | 60±11** | 41±8***^,♦^ | 8±4*** |
| *Spinal cord* | γ2I77 | 26±3 | 214±26^a^ | 4±2 |
|  | Pv-Δγ2 | 32±2 | 139±10** | 16±2*** |
|  | Pv-Δγ2-pr | 31±7 | 126±44** | 19±1*** |
| Cerebellum |  |  |  |  |
| *Granule cell layer* | γ2I77 | 47±11 | 37±4 | 13±3 |
|  | Pv-Δγ2 | 44±12 | 31±7 | 20±4 |
|  | Pv-Δγ2-pr | 44±20 | 51±32 | 27±15 |
| *Molecular layer* | γ2I77 | 21±7 | 13±3 | 1±2 |
|  | Pv-Δγ2 | 19±9 | 14±6 | 6±5 |
|  | Pv-Δγ2-pr | 25±16 | 11±4 | 5±5 |

Data are means ± SD, n = 5-6. *p < 0.05, **p < 0.01, ***p < 0.001 for the significance of the difference between the corresponding values for Pv-Δγ2 or Pv-Δγ2-rp and control γ2I77 mice; ^♦^p < 0.05, ^♦♦^p < 0.01, ^♦♦♦^p < 0.001 for the significance of the difference from the corresponding value for Pv-Δγ2 mice (one-way ANOVA and Newman-Keuls *post hoc* test). ^a^ For spinal cord samples, GABA concentration of 1 μM was used.
